# Serum and Cerebrospinal Fluid Levels of Transthyretin in Lewy Body Disorders with and without Dementia

**DOI:** 10.1371/journal.pone.0048042

**Published:** 2012-10-25

**Authors:** Walter Maetzler, Youyong Tian, Stephanie Maria Baur, Tina Gauger, Bartholomäus Odoj, Benjamin Schmid, Claudia Schulte, Christian Deuschle, Susanna Heck, Anja Apel, Arthur Melms, Thomas Gasser, Daniela Berg

**Affiliations:** 1 Center of Neurology, Department of Neurodegeneration and Hertie Institute for Clinical Brain Research, University of Tuebingen, Tuebingen, Germany; 2 DZNE, German Center for Neurodegenerative Diseases, Tuebingen, Germany; 3 Center of Neurology, General Neurology Department, University of Tuebingen, Tuebingen, Germany; 4 Department of Neurology, Nanjing First Hospital of Nanjing Medical University, Nanjing, China; University of Florida, United States of America

## Abstract

Parkinson’s disease (PD) without (non-demented, PDND) and with dementia (PDD), and dementia with Lewy bodies (DLB) are subsumed under the umbrella term Lewy body disorders (LBD). The main component of the underlying pathologic substrate, i.e. Lewy bodies and Lewy neurites, is misfolded alpha-synuclein (Asyn), and - in particular in demented LBD patients - co-occurring misfolded amyloid-beta (Abeta). Lowered blood and cerebrospinal fluid (CSF) levels of transthyretin (TTR) - a clearance protein mainly produced in the liver and, autonomously, in the choroid plexus - are associated with Abeta accumulation in Alzheimer’s disease. In addition, a recent study suggests that TTR is involved in Asyn clearance. We measured TTR protein levels in serum and cerebrospinal fluid of 131 LBD patients (77 PDND, 26 PDD, and 28 DLB) and 72 controls, and compared TTR levels with demographic and clinical data as well as neurodegenerative markers in the CSF. Five single nucleotide polymorphisms of the *TTR* gene which are considered to influence the ability of the protein to carry its ligands were also analyzed. CSF TTR levels were significantly higher in LBD patients compared to controls. Post-hoc analysis demonstrated that this effect was driven by PDND patients. In addition, CSF TTR levels correlated negatively with CSF Abeta_1–42_, total tau and phospho-tau levels. Serum TTR levels did not significantly differ among the studied groups. There were no relevant associations between TTR levels and genetic, demographic and clinical data, respectively. These results suggest an involvement of the clearance protein TTR in LBD pathophysiology, and should motivate to elucidate TTR-related mechanisms in LBD in more detail.

## Introduction

The term “Lewy body disorders (LBD)” subsumes the three entities Parkinson’s disease (PD) without dementia (PDND), PD with dementia (PDD), and dementia with Lewy bodies (DLB) [1]. In LBD, alpha-synuclein (Asyn)-positive Lewy bodies (LB) and Lewy neurites occur together with neurodegeneration [2].

Moreover, co-occurrence of amyloid-beta (Abeta) deposits with Asyn pathology is common, particularly in LBD patients with dementia [3], [4]. It is widely accepted that an Abeta_1–42_ production higher than its removal results in an enhanced presence of Abeta_1–42_ monomers, oligomers, insoluble fibrils and plaques in the central nervous system [5]. Age, *ApoE* genotype [6] as well as proteins and metabolites such as apolipoprotein E [7], apolipoprotein J (clusterin) [8], apolipoprotein A1 [9], uric acid [10], neprilysin [11], tau proteins [12], cystatin C [13], and transthyretin [14], [15] play a role in the neurotoxic aggregation of the usually soluble form of Abeta_1–42_. Similar mechanisms may lead to Asyn accumulation, at least for late onset PD although there is less evidence for this hypothesis [16].

The *transthyretin (TTR)* gene is located at chromosome 18q11 and consists of four exons [17]. TTR is mainly synthesized in the liver and the epithelial cells of the choroid plexus [18]. Recent results indicate that it is also a neuronal product up-regulated in Alzheimer’s disease (AD) [19]. It has binding sites for retinol binding protein (RBP), thyroxin and also for Abeta_1–42_ [20]. It can influence Abeta_1–42_ aggregation and destroy already formed Abeta_1–42_ fibrils by proteolytic cleavage of the peptides [21]. It has been suggested that TTR may be one of the major Abeta_1–42_-binding and -sequestering proteins in human cerebrospinal fluid (CSF) [14], [15]. Increased production of TTR in neurons exposed to Abeta_1–42_ toxicity, and reduced levels of the protein in blood [22], [23] and CSF [24]–[26] indicate an involvement of TTR (dys)function in clearance deficits as they occur in AD. In addition, there is very recent - at least indirect - evidence that TTR may be involved in Asyn clearance. Guerreiro and colleagues [27] observed highly increased levels of monomeric and oligomerized Asyn in the saliva of symptomatic heterozygous V30M *TTR* mutation carriers suffering from familial amyloidosis compared to healthy control individuals. Interestingly, Asyn levels in saliva of symptomatic *TTR* mutation carriers who underwent orthotropic liver transplantation (and, consecutively, had non-mutated *TTR*) were comparable with those of controls.

It has been demonstrated that not only the relatively rare mutations but also common variants of the *TTR* gene are differentially capable of altering the ability of TTR to carry and transport its ligands [28]. This has been shown in particular for the *TTR* single nucleotide polymorphisms (SNPs) rs13381522 and rs3764478 (both lie at the 5`end in the promoter region of the *TTR* gene), rs1800458 (located in exon 2, leads to a non-synonymous amino acid exchange of glycin to serin on position 26 of the *TTR* nucleotide sequence), rs723744 (localized in intron 3, does not result in amino acid differences), and rs36204272 (is intronic and spliced posttranslationally, should have no effect on the TTR protein) [15], [29]–[31]. As LBD patients obviously show neuronal and neural clearance deficits comparable to AD [16] and TTR seems to be critically involved in these mechanisms, we hypothesized that blood and CSF levels of TTR are altered in LBD patients and may be associated with clinical and demographic data, and that occurrence of the abovementioned SNPs are associated with these TTR levels.

## Study Participants, Material and Methods

### Ethics Statement

The study was approved by the ethics committee of the Medical Faculty, University of Tuebingen, Germany, and was performed according to the principles expressed in the Declaration of Helsinki. All participants gave their written informed consent. In case of compromised capacity of the participants to consent (MMSE <18 points), or another person was named to make decisions on behalf of the person, responsible persons consented on the behalf of the participant.

### Study Participants

Demented DLB, PDD and non-demented LBD patients (PDND), and elderly neurodegeneratively healthy control individuals (HC) were recruited from the ward and the outpatient clinic of the Neurodegenerative Department of the University of Tuebingen. LBD patients were diagnosed based on clinical criteria by specialists in the field of neurodegenerative movement disorders (DB, TG, WM). All PD patients fulfilled the UKPDS Brain Bank criteria [32]. PDD patients, in addition, met the Diagnostic and Statistical Manual of Mental Disorders-IV (DSM-IV) criteria for dementia as well as core and associated clinical features proposed by Emre and colleagues [33]. All DLB patients fulfilled the McKeith criteria for clinical diagnosis of probable DLB [34]. Control individuals underwent spinal taps for exclusion of vascular events or inflammatory conditions. Any clinical sign for a neurodegenerative disorder led to exclusion from the study.

All individuals underwent demographic analysis and clinical testing including a Hoehn&Yahr (H&Y) staging and a Mini-Mental State Examination (MMSE). Table 1 provides an overview of demographic, clinical, and biochemical data of the groups.

**Table 1:** Demographic, clinical, and routine biochemical data.

	LBD				Controls	*p*-value
	PDND		PDD	DLB		
Individuals (f/m) [n]	**131 (59/72)**				**72 (34/38)**	0.88
	77 (34/43)		26 (11/15)	28 (14/14)		
Age [years]: all	**70 (44–84)**				**57 (40–80)**	<.0001
	68 (44–81)		71 (62–84)	74 (50–83)		
females			**70 (44–84)**		**57 (43–79)**	**<.0001**
	68 (44–80)		71 (64–84)	76 (50–83)		
males			**69 (46–84)**		**58 (40–80)**	<.0001
	68 (46–81)		71 (62–84)	71 (58–78)		
Aao parkinsonism [years]	**63 (30–79)**					
	62 (30–79)		63 (51–74)	70 (49–78)		
Duration of parkinsonism[years]	**4.6 (0.1–25.0)**					
	4.1 (0.1–25.0)		8.7 (3.7–21.4)	2.9 (0.9–13.2)		
Aao dementia [years]	**69 (49–79)**					
		69 (58–79)		70 (49–79)		
Duration dementia [years]	**2.8 (0.4–12.2)**					
		2.1 (0.4–5.9)		3.4 (0.5–12.2)		
H&Y stage (1–5) [n]	**2.5 (1.0–4.5 )**					
	2 (1–4)		3 (2–5)	3 (1–4)		
MMSE (0–30)	**27 (10–30)**				**30 (27–30)**	<.0001
	29 (25–30)		21 (13–29)	19 (10–27)		
*ApoE4* [%]	**34**				**15**	0.047
	33	23		45		
CSF amyloid-beta_1–42_ [pg/ml]	**595 (134–1458)**				**907 (264–1446)**	<.0001
	629 (134–1458)	497 (147–851)		453 (228–1241)		
CSF total tau [pg/ml]	**199 (25–1074)**				**183 (32–868)**	0.56
	176 (25–1074)	254 (48–660)		236 (77–596)		
CSF phospho-tau [pg/ml]	**40 (12–127)**				**39 (14–158)**	0.39
	36 (15–127)	57 (12–109)		50 (22–86)		

Demographic, clinical, and biochemical data of patients with Lewy body disorders (LBD) and controls, presented with median (range). *P*-values were determined using the Wilcoxon rank sum test, or the Fisher’s exact test. Aao, age at onset; *ApoE4, Apolipoprotein E4*; CSF, cerebrospinal fluid; DLB, dementia with Lewy bodies; f, female; H&Y, Hoehn&Yahr stage; m, male; MMSE, Mini-Mental State Examination; PDD, Parkinson’s disease with dementia; PDND, Parkinson’s disease non-demented.

### Cerebrospinal Fluid and Serum Collection

CSF and serum collection as well as determination of routine diagnostic parameters were performed according to standardized protocols (for details see [35]). We only used samples specifically dedicated to this project from our biobank (http://www.hih-tuebingen.de/de/nd/biobank/for-researchers/), without any previous freeze-thaw cycle. Our biobank fulfills highest international standards with regard to collection, preparation and storage of biosamples. Samples for determination of TTR levels were centrifuged (CSF: 4000 g, 4°C, 10 min; blood: 2000 g, 4°C, 10 min) and stored at −80°C within 60 minutes after collection until analysis. Only samples of patients with normal routine CSF diagnostics were included in the study, slightly increased CSF albumin levels (up to 450 mg/L) were tolerated.

### TTR, Abeta_1–42_, Total Tau and Phospho-tau Measurement

TTR levels in CSF and serum were measured using a laser nephelometric method with Ics Pab Prealbumin Reagent Test Kit on Beckman Coulter IMMAGE® Immunochemistry Systems according to manufacturer’s instructions (Beckman Coulter GmbH, Krefeld, Germany). Samples were run in triplicate. We observed within run precision of <2.0% coefficient of variation (CV), and total precision of <5% CV.

CSF Abeta_1–42_, total tau and phospho-tau levels were determined using commercially available ELISA kits (Innogenetics NV, Ghent, Belgium).

### TTR and ApoE Genotyping

Genomic DNA was extracted using standard protocols. The coding sequence of the *TTR* gene was determined with Sanger-sequencing technique to identify the SNPs rs1800458 and rs36204272. SNPs rs13381522, rs3764478, and rs723744 which are located at the *TTR* intron or promoter region were analyzed by the SNaPshot method. Briefly, a PCR with specific SNaPshot primers was performed. After single base extension with fluorescent-labeled ddNTPs the SNaPshot product was analyzed by capillary gel electrophoresis with ABI Prism 3100 Genetic Analyzer sequencer (Applied Biosystems Life Technologies GmbH, Darmstadt, Germany). Fluorescence data were analyzed using Gene Mapper v3.5.

*ApoE* genotypes were obtained by PCR amplification of exon 4 of the *ApoE* gene, and subsequent digestion with enzyme HhaI according to [36]. All SNPs investigated were in Hardy-Weinberg equilibrium.

### Data Analysis

Data were analyzed with JMP software (version 9.0.2, SAS). As dementia associated with LBD may per se be a relevant factor associated with TTR levels, PDD and DLB were not only considered as single groups but also comprised as a PDD/DLB group (see Table S1 for details). Demographic and clinical data were analyzed with the Wilcoxon signed rank test (LBD versus controls; presented as median and range) or Fishers exact test. As age was significantly different between LBD and controls and as there is some evidence that serum TTR may be associated with age [37], we corrected both serum and CSF TTR levels for this covariate using a regression model. The coefficient of determination (r^2^) was used as a measure for the proportion of response variation which was explained by the regressors in the model. Significance of each model effect was assessed by the likelihood ratio.

Differences were considered significant at *p*<0.05. Post-hoc analyses (TTR levels of PDND versus controls, PDND versus demented LBD, controls versus demented LBD) were corrected for multiple testing (see Table 2 and Table S1 for details).

**Table 2:** Transthyretin values of the investigated cohorts.

		LBD			Controls	*p*-value
		PDND	PDD	DLB		
CSF TTR [mg/dl]		**1.73 (0.58–2.84)**			**1.58 (0.58–2.61)**	0.037
		1.80 (0.58–2.32) ^§^	1.60 (0.58–2.84)	1.68 (0.58–2.68)		
Serum TTR [mg/dl]	females	**25.3 (12.1–39.8)**			**27.3 (19.6–48.1)**	0.39
		26.7 (16.1–39.8)	21.9 (15.5–30.1)	20.8 (12.1–31.7)		
	males	**29.7 (15.2–46.5)**			**31.3 (19.9–43.9)**	0.54
		31.2 (17.0–46.5)	25.6 (15.2–35.1)	30.9 (21.7–37.1)		

Transthyretin (TTR) levels were calculated using a regression model with age as a covariate. As serum TTR levels were significantly different between females and males (see text) these values were calculated separately. *P*-values <0.05 between patients with Lewy body disorders (LBD) and controls were considered significant. Post-hoc analyses were performed for comparison of TTR levels between the following cohorts: Parkinson’s disease non-demented (PDND), Parkinson’s disease with dementia (PDD), dementia with Lewy bodies (DLB), controls, PDD versus DLB, PDD versus controls, and DLB versus controls, with *p-*values <0.01 (0.05/5 groups) considered significant. CSF, cerebrospinal fluid.

## Results

### TTR Levels and Demographic Variables

TTR values in CSF and serum were normally distributed, and did not correlate. They were not age-related, neither in CSF (controls, r^2^ = 0.02, *p* = 0.88; LBD patients, r^2^ = 0.01, *p* = 0.64) nor in serum (controls, r^2^ = 0.01, *p* = 0.19; LBD patients, r^2^ = 0.02, *p* = 0.11). CSF TTR values were approx. 18-fold lower than serum values (1.7 versus 30.1 mg/dl).

Gender did not differ significantly between LBD patients and controls. CSF TTR values were comparable between females and males (controls, *p* = 0.56; LBD patients, *p* = 0.59). Serum TTR levels were significantly higher in males than in females (controls, *p* = 0.03; LBD patients, *p* = 0.002) and were therefore calculated separately.

### TTR Levels and LBD-associated Parameters

An overview is given in Table 2. CSF TTR values were significantly higher in LBD patients (1.73 mg/dl) compared to controls (1.58 mg/dl). The post-hoc analysis demonstrated that, within the LBD group, PDND patients accounted for this effect (1.80 mg/dl, r^2^ = 0.07, p = 0.008 compared to controls). Serum TTR levels were not significantly different between LBD patients and controls, and among LBD patients (see also Table S1).

CSF and serum TTR values were neither significantly associated with clinical parameters (age at onset of parkinsonism, duration of parkinsonism, Hoehn&Yahr stage, age at onset of dementia, duration of dementia, MMSE score), nor with *ApoE* status.

### TTR Levels and Neurodegenerative Markers

There were weak but significant negative correlations between CSF TTR values and respective levels of CSF Abeta_1–42_ (r^2^ = 0.03, *p* = 0.02), CSF total tau (r^2^ = 0.03, *p* = 0.02) and CSF phospho-tau (r^2^ = 0.03, *p* = 0.03). No significant correlations were detectable between serum TTR values and neurodegenerative markers.

### TTR Genotypes

None of the analyzed *TTR* SNPs (rs723744, rs13381522, rs3764478, rs1800458 and rs36204272) showed a significant association with LBD occurrence. In addition, no relevant associations of the SNPs with demographical, clinical and biochemical parameters were detectable (data not shown).

## Discussion

In this study investigating TTR values in blood and CSF of LBD patients, CSF TTR levels were significantly higher in this cohort of patients compared to controls. The effect was mainly driven by non-demented LBD patients, explaining, as a diagnosis, approximately 7 percent of the variation in the CSF TTR levels. This makes it tempting to speculate that Asyn pathology alone (PDND patients rarely have marked additional brain pathology such as leucoaraiosis [38], [39] and Abeta_1–42_ pathology [4], [23], [40]) induces expression of central TTR. This observation may be explained by the known occurrence of oxidative stress in LBD [35], [41], as CSF TTR values have been observed to rise in response to oxidative stress [14]. In addition, an involvement of TTR in inflammatory processes - a common feature observed in brains of patients suffering from LBD - has also been suggested [42]. In the light of previous reports which mainly found *decreased* CSF TTR levels in disorders that are not primarily associated with inflammation (i.e., depression [43], AD [14], [24] and amyotrophic lateral sclerosis [44]), but *increased* levels in disorders typically associated with inflammatory processes (such as Guillain Barré syndrome and chronic inflammatory demyelinating polyneuropathy [45]–[47]) it may be interesting to focus in future studies on putative interaction pathways between inflammation and TTR in Asyn-associated pathology.

Actually it is not entirely clear whether (misfolded) Asyn is a target protein of TTR. The latter mainly functions as a transport protein, and is able to transfer e.g. Abeta_1–42_, as a misfolded peptide, from the neuron to the CSF [25]. A recent study also showed that Abeta_1–42_-stressed neurons increase the expression of TTR [48]. Thus it is intriguing to hypothesize that Asyn is also a target protein of TTR, and TTR may have an influence on Asyn-associated pathology. Indeed, indirect evidence for this suggestion comes from a recent study investigating levels of monomeric and oligomeric Asyn in saliva using MALDI-TOFTOF MSMS ion search and Western blotting [27]. In this study, symptomatic individuals with a heterozygous V30M mutation in *TTR* had much more intense Asyn-positive bands and a higher number of such bands at higher molecular mass (indicative of oligomeric forms of Asyn) than had control individuals and, most interestingly, *TTR* mutation carriers who underwent orthotropic liver transplantation. This strongly indicates that TTR is associated with clearance of (misfolded) Asyn.

Interestingly, demented LBD patients had relatively low CSF TTR levels, comparable to controls [49]. This is exciting in the light of recent studies demonstrating decreased CSF TTR levels in AD patients [14], [24], and the regular occurrence of Abeta_1–42_ pathology in demented LBD patients. Together with the observation that PDND patients have increased CSF TTR levels one may hypothesize that demented LBD patients face two driving forces with regard to TTR-associated pathways: Asyn pathology which goes along with increased TTR expression, and Abeta_1–42_ pathology associated with decreased TTR expression. Based on the abovementioned associations between TTR levels and amyloid pathology, one can also hypothesize that increased TTR levels in the brain protect against Abeta_1–42_ pathology. Indeed, there is accumulating evidence from *in-vitro* and *in-vivo* studies that TTR has beneficial direct and indirect effects on Abeta-associated pathology (reviewed in [14]).

Serum TTR levels were not significantly different between LBD patients and controls. Post-hoc analyses demonstrated slightly reduced TTR levels in demented compared to non-demented LBD patients. Although results did not survive correction for multiple testing, they may motivate to future, hypothesis-driven studies focusing on this particular aspect as decreased serum TTR levels have already been described in AD patients [22], [23], [50],, and are regarded as a biochemical marker for malnutrition [25], [51] and inflammation [52].

We found weak but significant negative correlations between CSF TTR levels and the three actually best-established neurodegenerative markers in CSF, i.e. Abeta_1–42_, total tau and phospho-tau. Together with the observed lack of such an association between peripheral TTR and these neurodegenerative markers, the observations may point to a specific interaction of central TTR with LBD-associated amyloid clearance. Higher CSF TTR levels were observed in non-demented LBD patients who regularly show relatively low CSF tau levels [53], [54]. This argues for an interaction of TTR with the specific neurodegenerative process (total tau) and axonal pathology (phospho-tau). The negative correlation between TTR and Abeta_1–42_ CSF levels is somehow counterintuitive and more difficult to explain as, in individuals with Abeta_1–42_ pathology, CSF Abeta_1–42_ is regularly low and tau parameters are high. Under physiological conditions, CSF TTR binds Abeta_1–42_ and keeps it soluble especially in CSF [25]. Decreased CSF TTR values may indicate that this dynamic equilibrium is affected, leading to accumulation and aggregation of Abeta_1–42_ proteins, amyloid formation, and neurotoxicity [15].

Analysis of SNPs that have been shown to influence binding capacity of TTR to amyloid did not add relevant information in this study. This may be due to sample size, but also to the fact that rather the absolute quantity of TTR than its function may be associated with LBD pathophysiology. Interestingly, we also did not find a significant association between demographic and clinical parameters (except the above reported, diagnosis of LBD and occurrence of dementia), and TTR values. This may indicate that TTR, although obviously involved in amyloidogenic and clearance pathways in LBD, cannot serve as a marker of disease progression and severity.

In conclusion, data presented in this study argue for a role of TTR in both Asyn- and Abeta_1–42_-associated LBD pathologies. The obvious interaction of centrally produced TTR with pathophysiological mechanisms in these neurodegenerative processes should motivate to more in-depth analyses.

## Supporting Information

Table S1 Post-hoc analyses of transthyretin levels between different groups
(DOC)
