# Supplementary material for: Serum and Cerebrospinal Fluid Levels of Transthyretin in Lewy Body Disorders with and without Dementia
Source: PLoS One. 2012 Oct 25;7(10):e48042. doi: 10.1371/journal.pone.0048042 (PMC3485000; doi:10.1371/journal.pone.0048042)
Supplement: Table S1 — Post-hoc analyses of transthyretin levels between different groups (DOC) [file pone.0048042.s001.doc]

**Table S1: Post-hoc analyses of transthyretin levels between different groups**

| CSF TTR levels |  | p-value |
| --- | --- | --- |
|  | PDND versus PDD | 0.29 |
|  | PDND versus DLB | 0.33 |
|  | PDND versus PDD/DLB | 0.33 |
|  | PDND versus controls | **0.008** |
|  | PDD versus DLB | 0.83 |
|  | PDD versus controls | 0.47 |
|  | DLB versus controls | 0.29 |
|  | PDD/DLB versus controls | 0.57 |
| Serum TTR levels (females) |  |  |
|  |  |  |
|  | PDND versus PDD | 0.06 |
|  | PDND versus DLB | 0.05 |
|  | PDND versus PDD/DLB | 0.03 |
|  | PDND versus controls | 0.60 |
|  | PDD versus DLB | 0.97 |
|  | PDD versus controls | 0.09 |
|  | DLB versus controls | 0.08 |
|  | PDD/DLB versus controls | 0.07 |
| Serum TTR levels (males) |  |  |
|  | PDND versus PDD | 0.03 |
|  |  |
|  | PDND versus DLB | 0.85 |
|  | PDND versus PDD/DLB | 0.14 |
|  | PDND versus controls | 0.92 |
|  | PDD versus DLB | 0.03 |
|  | PDD versus controls | 0.11 |
|  | DLB versus controls | 0.63 |
|  | PDD/DLB versus controls | 0.57 |
|  |  |  |

P-values were calculated using a regression model with age as a covariate (see statistics section for details). P-values < 0.01 (0.05/5 groups: controls, dementia with Lewy bodies (DLB), Parkinson’s disease with dementia (PDD), PDD/DLB, Parkinson’s disease non-demented (PDND)) were considered significant. CSF, cerebrospinal fluid; TTR, transthyretin.
